# The emergence and regression of political priority for refugee integration into the Jordanian health system: an analysis using the Kingdon’s multiple streams model

**DOI:** 10.1186/s13031-024-00588-3

**Published:** 2024-04-15

**Authors:** Shatha Elnakib, Laila Akhu-Zaheya, Wejdan Khater, Lama Bou-Karroum, Gladys Honein-AbouHaidar, Sabine Salameh, Yusra Ribhi Shawar, Paul Spiegel

**Affiliations:** 1 Johns Hopkins Bloomberg School of Public Health, Baltimore, MD USA; 2 Faculty of Nursing, Jordan University of Science and Technology, Irbid, Jordan; 3 American University of Beirut, Beirut, Lebanon; 4 Paul H. Nitze School of Advanced International Studies, Washington, DC USA

**Keywords:** Refugee integration, Jordan, Health system, Kingdon’s multiple streams model, Syrian refugees

## Abstract

**Background:**

The prolonged presence of Syrian refugees in Jordan has highlighted the need for sustainable health service delivery models for refugees. In 2012, the Jordanian government adopted a policy that granted Syrian refugees access, free of charge, to the national health system. However since 2012, successive policy revisions have limited refugee access. This paper seeks to understand factors that initially put refugee integration into the health system on the policy agenda, as well as how these same factors later affected commitment to sustain the policy.

**Methods:**

This paper draws on data from a document review of 197 peer-reviewed and grey literature publications, a media analysis of newspaper articles retrieved from four officially recognized newspapers in Jordan, and 33 semi-structured key informant interviews. We used Kingdon’s Multiple Streams Model – a well-established tool for analyzing policy adoption – to understand how political priority developed for integration of refugees into the health system.

**Results:**

We find that several factors helped bring attention to the issue, namely concerns over infectious disease transmission to host communities, high rates of chronic conditions among the refugee population and the increasingly urban and dispersed nature of refugees. At the outset of the conflict, the national mood was receptive to refugees. Politicians and government officials quickly recognized the crisis as an opportunity to secure material and technical support from the international humanitarian community. At the same time, global pressures for integrating refugees into national health systems helped move the integration agenda forward in Jordan and the region more broadly. Since 2012, there were several modifications to the policy that signal profound changes in national views around the continued presence of Syrian refugees in the country, as well as reduced external financial support which has undermined the sustainability of the policy.

**Conclusion:**

This case study underscores the dynamic nature of policymaking and the challenge of sustaining government commitment to the right to health among refugees. Our analysis has important implications for advocates seeking to advance and maintain momentum for the integration of refugees into national health systems.

**Supplementary Information:**

The online version contains supplementary material available at 10.1186/s13031-024-00588-3.

## Introduction

For the first time on record, 100 million people - comprising over 1% of the global population – were reported to be living in a state of forced displacement [1]. Of those, 83% are hosted in low- and middle-income countries [2]. Why refugees are predominantly hosted in developing countries in part stems from the lack of resources crisis-affected people need to flee beyond neighboring countries, but can also be explained by historical and political relationships, as well as other sociopolitical dynamics. Contemporary refugee crises are becoming more protracted and intractable, and the vast majority of refugees live in long-term circumstances [3]. The Middle East is home to several prolonged crises, with the most recent Syria crisis drawing global attention. Overall, the Middle East region hosts 2.4 million refugees, 12.6 million internally displaced people (IDPs), 251,800 asylum seekers and 370,300 stateless persons [1]. The Syria crisis alone has resulted in the displacement of 6.5 million people [1]. By the end of 2015, Syria’s neighbors – Turkey, Lebanon, and Jordan – had hosted 27% of the global refugee population [4]. Eleven years since the onset of the crisis, the same three countries continue to host the overwhelming majority of Syrian refugees. As of October 2023, Turkey hosts over 3 million Syrians, Lebanon hosts 789,000, and Jordan hosts 652,000 refugees and asylum seekers.

The magnitude of the refugee crisis and the prolonged nature of conflicts at the root of many refugee crises have highlighted the acute need for durable and long-term solutions [4]. In the health sector, the question of how to deliver health services sustainably in light of the increasingly protracted nature of refugee crises, while not new, has received renewed attention. In recent years, the humanitarian-development-peace nexus has been increasingly cast as a solution to problems posed by protracted refugee crises, and integration of refugees into national health systems has emerged as a popular solution [5]. However, integration is a politically loaded issue because it requires host governments to acknowledge and accept the prolonged presence of refugees in the country, which many are reluctant to do.

Jordan presents a case study where integration into health systems was adopted as a national policy in 2012 in reaction to the mass displacement of Syrian refugees into the country. Since, then several policy changes have taken place. As of 2023, Jordan hosts around 650,000 registered Syrian refugees, but the total including unregistered refugees is estimated to be 1.1 million Syrians. This is a significant proportion of the country’s population of 11 million [6].

This paper seeks to identify factors that contributed to the emergence of national priority for refugee health integration in the country and chronicle modifications that have occurred, which reflect profound changes in Jordan’s national and government views and capacity to respond to the refugee crisis. While the country hosts other refugees, including Iraqi and Palestinian refugees, this analysis focuses on the response to the Syria crisis which ushered a larger number of refugees into the country compared to the Iraq influx, and was more recent compared to the Palestinian crisis. Drawing on Kingdon’s Multiple Streams Model – a well-established approach for analyzing policy adoption – our analysis seeks to shed light on enablers of integration in this setting, as well as factors that have undermined the integration agenda [7]. The analysis has important implications for advocates seeking to advance the integration of refugees into national health systems in protracted crisis settings.

## Methods

This study draws on a document review and qualitative data collected among key informants with the aim of understanding the evolution of policies on refugee integration into health systems in Jordan and the factors that contributed to the prioritization of the issue. We define the refugee integration agenda loosely as concerted efforts to provide healthcare access to refugees via national institutions and systems, but acknowledge the definitional challenges in relation to integration and the lack of operational guidance on what exactly it constitutes. The analysis focuses on the adoption of a 2012 policy that integrates Syrian refugees into the national health system and chronicles changes that subsequently occurred in the health integration agenda in the country.

A. Overview of the Jordanian Health System

The Jordanian healthcare system consists of public/semi-public and private sectors, with the public sector accounting for majority of hospital beds [8]. The Ministry of Health (MoH) finances and administers the public sector. (6) Together, both sectors include hospitals, primary care centers, and other medical services. Primary health care clinics are located in urban and rural areas and range from small, specialized clinics to comprehensive multi-clinic centers. These provide a range of services including maternal and child healthcare, vaccinations, and chronic disease care. Because of the presence of a sizeable Palestinian refugee population in the country, health services to Palestinian refugees are provided through UNRWA-supported clinics, with more than 1.1 million Palestinian refugees served by UNRWA clinics [9]. Palestinian refugees requiring hospital care are reimbursed for costs incurred for inpatient care at public and private facilities by the organization. With the Syria crisis, the United Nations High Commissioner for Refugees (UNHCR) has also been delivering health services inside camps to Syrian refugees [8]. The situation for out-of-camp refugees is more complex, with the Ministry of Health providing the bulk of support through its public services. Policies on access to health services for out-of-camp Syrian refugees have changed over time as explained later in the paper.

B. Document Review

We undertook a review of peer-reviewed and grey literature as well as news media articles published in the period between 2011 to December 2021, to capture literature that is relevant to the three policies featured in this analysis. Articles were selected based on their relevance to political priority development for refugee integration in the country. We searched a total of three electronic databases: Medline, PubMed and Scopus using a broad search strategy to maintain breadth of coverage (available in Supplementary File 1). Grey literature was identified through a targeted search of publications and reports posted on websites of United Nations (UN) and international organizations, and government agencies. We relied on additional snowballing using the bibliographies of publications mapped and sought recommendations for publications from key stakeholders including a resource person from the Jordanian Ministry of Health who is in charge of the Syria crisis response. Articles were included if they discussed the situation of Syrian refugees in Jordan. Articles that addressed the situation of Syrian refugees in other countries, including in Syria, were not eligible for inclusion. We also excluded studies that addressed the situation of refugees in Jordan who were not Syrian. In terms of focus, we included articles that had a health focus, such as health care access, health care services, health care delivery, health care system, health care utilization, health needs, and health care workers. Articles that did not have an exclusive health focus were still eligible for inclusion if they had a health focus and in extracting data from thes articles, studies teams focused on health-relevant aspects.

**Fig. 1:**
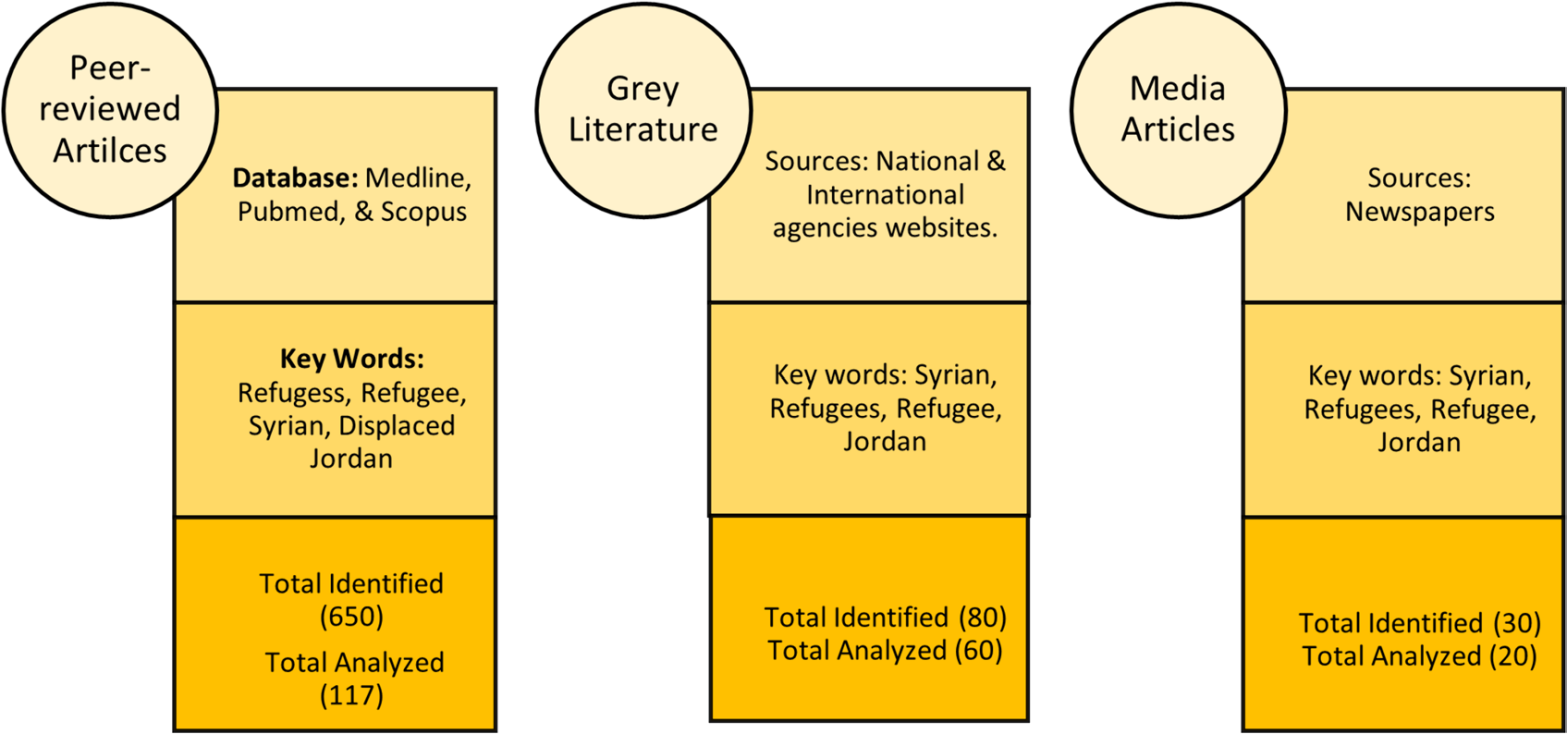
Document review data sources

In total, we retrieved 760 relevant articles, of which 197 were eligible for inclusion and review (Fig. 1). The Kingdon’s Multiple Streams Model was used to guide the development of a standard data extraction sheet, enabling an understanding of factors driving the adoption of policies around refugee integration in Jordan. Findings from the document review were summarized in a short report and were then compared against findings from the key informant interviews during an analysis workshop that took place in Amman with the in-country team.

C. Key informant interviews

Based on the document review, we identified individuals who possessed knowledge about policies on refugee access to health services or who were centrally involved in policy development around integration of refugees into the Jordanian health system. A total of 33 face-to face semi-structured interviews were conducted, the majority of which in Arabic, between January 2018 and March 2019 (Table 1). Interviews lasted between 45 and 60 minutes. Sample size was guided by extent to which theoretical saturation was reached. Interview guides are available in Supplemental file 2.

**Table 1:** Key informants and their affiliations

Type of organization/participant	*N* = 33
Government agencies (MoH, health directorates, High Health Council)	5
International and national non-governmental organizations	4
Health providers	
Working in PHCs	8
Working in Hospitals	12
Hospital/facility managers	4

Interviews were audio recorded, transcribed in Arabic or English and transcripts were analyzed using content analysis methods. A combination of deductive and inductive codes was used. Deductive codes were based on Kingdon’s Multiple Streams Model and fell into one of three categories: problem, policy and political. Additional sub-codes emerged under these parent codes based on ideas and themes that emerged from the empirical data. Initial line-by-line codes were consolidated into more focused codes which were applied to the data using the data management software Quirkos [10]. Three researchers, all native Arabic speakers and with graduate level training in public health, coded the data. Coding was iterative and involved continuous refinement of the codebook along with regular check-ins for consistency. Following coding, a two-day analysis workshop took place where all the coded data was reviewed by the first, second, third and fourth authors. The workshop allowed for discussion of the data from both the key informant interviews and document review and the themes arising from them, and enabled the researchers to draw comparisons across time, participants’ roles and facilitated triangulation of findings. The team spent time discussing the relevance of findings from the interviews to those from the document review, corroborated findings across the different data sources, and tried to explain discrepancies that arose through discussion and follow-up interviews.

D. Historical Narrative

In addition to analyzing data from the document review and key informant interviews, we constructed a timeline that chronicles key events leading to policy changes on refugee access to health services in the country. This was based on a comprehensive and chronological document and media review in which we scanned relevant articles on the websites of four officially recognized newspapers (Jordan News Agency (Petra); Alrai, Addustour and Al Ghad). The media review aimed to elucidate the development and implementation of policies on refugee integration in the health system and to assess the national mood impacting policymaking and discourse. It also helped bring to light focusing events and social, political factors impacting policies around integration.

E. Analytical framework

Kingdon’s multiple streams framework explains agenda setting by proposing the emergence and convergence of three independent streams – problems, politics and policies. While formulated in the context of the United States, the model has been applied in various other settings to understand the emergence of policy priorities and agenda-setting processes [11].

The first stream – termed the *problem stream* - concerns how the problem is perceived and understood by the public [7]. When an issue is elevated to problem status, government action is seen as needed to resolve it. Key events help galvanize awareness of the problem and put it on the agenda of policymakers. These include key indicators, which paint a picture of the gravity of a particular situation, and focusing events or dramatic occurrences and developments that attract public attention such as disasters or crises.

The *political stream* depicts the general political national mood that impacts agenda-setting and prioritization [7]. This includes the orientation and interests of the ruling regime, politicians, parliamentarians, UN agency heads among others and the overarching macro-environment impacting policymaking.

The *policy stream* represents the process by which policy solutions come about and why and how one or more solutions get chosen from a set of alternatives [7].

While operating independently, two or more streams must come together for a window of opportunity to open [11]. At this point, an issue emerges on the national agenda.

## Ethical approval

The study protocol was reviewed by the Institutional Review Board of Jordan University of Science and Technology and Johns Hopkins University in Baltimore, MD, USA. The former provided approval (IRB7/123/2019) and the latter provided an exemption owing to the minimal risks posed by the research.

## Results

**Fig. 2:**
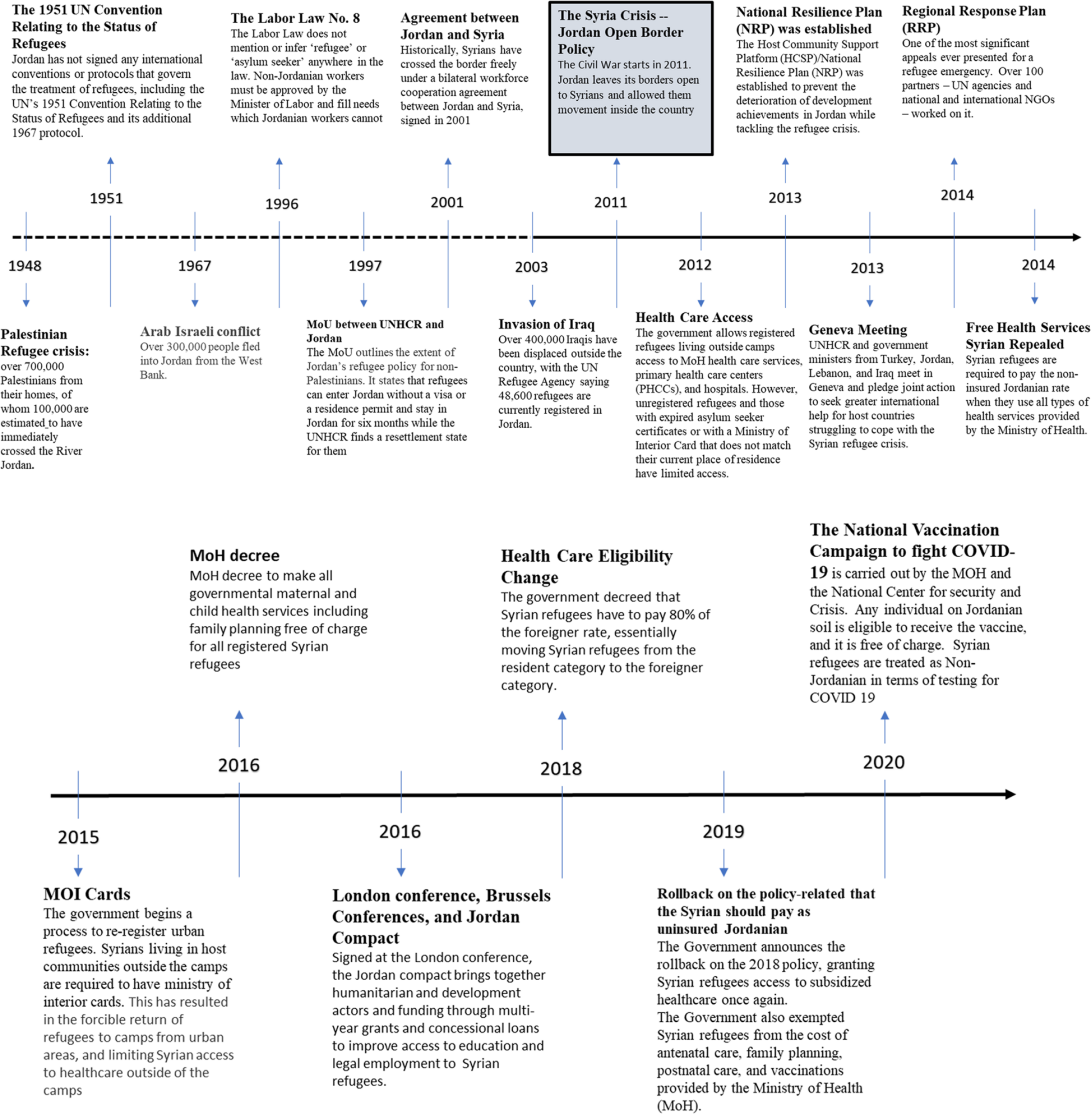
Timeline of key events relevant to policies on healthcare access to Syrian refugees

Figure 2 illustrates a timeline of key events and developments leading up to the formulation of policies relevant to the integration of refugees into the Jordanian health system. As illustrated in the timeline, since the onset of the Syria crisis, three key policies came into effect which had important implications for Syrian refugees’ access to healthcare in the country. Through this case study, we examine factors and events shaping the adoption of these policies which highlight two patterns: prioritization of integration of refugees into social structures and systems, and specifically into the national health system and subsequent ebbs and flows in government support for health integration. The first period spans 2011–2014 and the second spans 2014 to the present. We use the Kingdon’s Multiple Streams Model to understand how factors at the problem, political, and policy stream converged to set the stage for the adoption of health integration in 2012. We then attempt to explain policy reversal that occurred later using elements from the same Model.

## Problem stream

The issue of refugee health service delivery in Jordan quickly acquired problem status following the first wave of the Syrian influx. The sheer number of refugees who crossed the border in the first year of the crisis, their concentration in urban areas along with real and perceived threats of infectious disease outbreaks and high rates of non-communicable diseases in the refugee population put the problem on the policy agenda.

### Large and sudden inflow of Syrian refugees into Jordan and increasingly urban concentration of the refugee population

The Syria conflict saw the mass displacement of Syrian nationals into neighboring countries, with March 2011 signaling the beginning of the influx. Jordan quickly became the third largest host of Syrian refugees in the region, with estimates ranging between 660,000 to 1.26 million Syrians residing in the country representing a 10% population increase [12]. Despite a legacy of receiving refugees from neighboring countries, the magnitude of the Syrian inflow was unprecedented, and according to participants “abrupt” and “unexpected” which helped elevate the issue of refugee healthcare delivery to problem status. The scale and pace of displacement of Syrians into Jordan created a sense of urgency to address the consequences of the influx and the emerging healthcare needs of refugees.

**Fig. 3:**
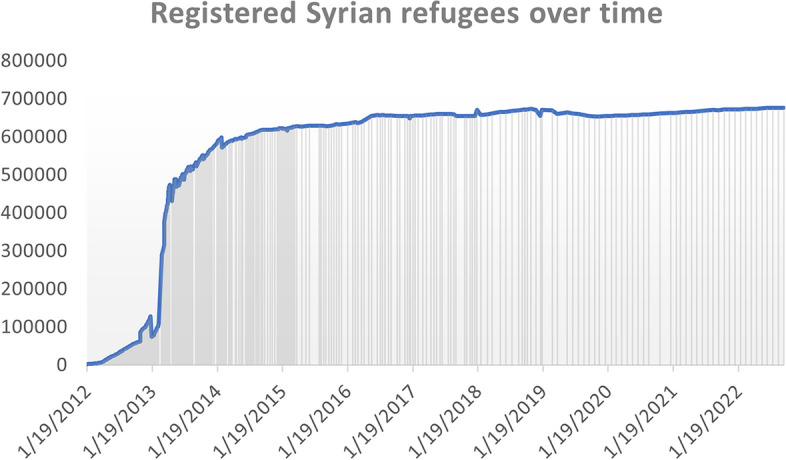
Trend in number of registered Syrian refugees over time (2012–2022)

According to UNHCR data, in 2012 alone, 117,321 refugees had been registered in the country, and by the end of 2013 the number had almost quadrupled (Fig. 3).

Participants noted that the features of the Syria crisis made it hard for the government to absorb and address the health needs of the refugee population. While Jordan established refugee camps like Za’atari, there was a reluctance to establish too many camps after the country’s experience with Palestinian refugees who were integrated into Jordanian society. Moreover, the majority of refugees were located outside of camps, dispersed widely across urban and peri-urban areas which precluded the introduction of parallel health service delivery structures. This was seen as key in setting the stage for the integration of refugee healthcare within the national health system.

### Emergence (and reemergence) of new infectious diseases

Another key factor that painted the refugee crisis as a matter requiring urgent public health intervention was the occurrence of infectious disease outbreaks in the country in 2012 and 2013. Attributed to the refugee influx, outbreaks were perceived as threatening to reverse gains made over decades of investment in the Jordanian health system. They also sparked fears about recurrence of diseases that were once eradicated as well as concerns around disease transmission to the local host population [13]. In 2013, after 3 years of zero measles cases, an outbreak occurred among Syrian refugees and over 200 cases were reported [14–16]. Other outbreaks included tuberculosis and cutaneous leishmaniasis. These outbreaks spurred sustained media coverage and fear in the host population [17–20]. As articulated by participants:

“The spread of infectious diseases such as scabies and tuberculosis because of the conditions in which they were living was a huge consideration. Rent prices had increased, so Syrian refugees would live in overcrowded and unsanitary conditions with high humidity and poor ventilation, which increased risks of infectious disease.” Healthcare provider in Mafraq

“Their disease profile was different. We had forgotten about some of these diseases that re-emerged with their arrival like Tuberculosis.” Healthcare Provider in Amman

“The Syrians have become part and parcel of the Jordanian population, citizens like us, they are present alongside us, and intermixed with us. As a result their health matters because their health reflects on our health. I always say that the situation it is like a time bomb, that can explode anytime, so we have to make sure their presence doesn’t jeopardize us” Policymaker

### High rates of chronic diseases

While infectious diseases prompted fears around transmission to the host community, the high rates of non-communicable diseases (NCDs) in the refugee population raised additional concerns that if left untreated, hospitalization rates would increase, posing a burden on the health system and overstretching services. As one health provider in Amman stated “*What we noticed from the refugees who started coming to our facilities is that so many of them had a chronic disease. This group has high prevalence of chronic conditions and mostly undiagnosed.”*

The high prevalence of chronic diseases, which was unusual among refugee populations who typically hail from countries that have not undergone the demographic and epidemiological transitions, necessitated a shift in humanitarian response, from vertically organized services to local health system strengthening. The country’s experience with Iraqi refugees who were similarly unique in their demographic and epidemiological profile had contributed to these concerns. In tandem, there was growing global attention to the issue of non-communicable diseases among refugee populations which highlighted the urgency of the situation. Pre-crisis data on rates of chronic diseases among Syrians indicated a high burden of obesity, diabetes and coronary heart disease, and decreasing levels of infectious disease [21]. While not fully available at the beginning of the crisis, empirical data on the prevalence of chronic diseases slowly painted a picture of the severity of the burden of NCDs, and eventually national representative surveys done in Jordan such as the 2014 Syrian refugee health access survey revealed that half of the refugee population had at least one member of their household living with at least one non-communicable disease [22, 23]. Newspaper and media outlets shone a light on the severity of the issue with headlines such as “Syrian refugees fight cancer without ‘weapons,’” and “Syrian refugees and the double burden” in reference to Syrian refugees’ simultaneous experience of displacement along with a rising burden of non-communicable diseases [24, 25].

## Political stream

### National mood at the outset of the crisis

The initial reaction of the Jordanian government to the Syrian influx was welcoming as evidenced by the open border policy adopted between 2011 and 2013 which allowed Syrian refugees entry into the country. Media coverage was also largely sympathetic to the plight of Syrian refugees, and public opinion was initially in favor of opening the borders to refugees [26–28]. As expressed in a news article published in April 2012:

“The position of the king, the government and the people towards the Syrian people is first and foremost dictated by a humanitarian imperative. Helping those affected by the Syria crisis is our national duty and legal obligation - dictated by ties of blood, kinship, and lineage, not to mention obligations arising from our historical ties and geographic proximity.” Alrai news article (21)

A confluence of factors contributed to the favorable reaction, including social ties and kinship between Jordanians and Syrians that predated the conflict, a shared religious and cultural identity, and as well as feelings of religious obligation.

“These Syrians were from the border villages they were poor and vulnerable, and they don’t have anyone but us to help them. And many of them were related to us by blood. Especially those in places like Daraa, Ramtha, As-Sarih. I for example have many relatives in Daraa in Syria. My cousin came from Daraa and resided with us with her son.” Policymaker

“For the Jordanian people, acceptance of the Syrian influx stemmed from the social bond that exists between the two groups, we are bound by ties that go far back in time, hundreds of years back. You will see that some of us belong to the same clan, some members of the clan live here in Jordan and others in Syria. So when the conflict erupted, members of the clan here would help their relatives in Syria. So Syrians were able to impose their presence in Jordan through these ties. In Ramtha, for example, you will see the two communities are very much integrated, intermarriages are very common, and so the refugee community is completely integrated in every respect.” Hospital manager, Alramtha

### The appeal of material and technical support from the international community

Jordan’s status as a host country in the Syria crisis helped it secure substantial material and technical support from the international humanitarian community, which helped it advance its own national development. Indeed, international aid pouring into Jordan on the heels of the Syria refugee crisis made the country one of the top recipients of foreign aid in the world [29, 30]. According to some participants, despite the pressure the influx placed on the country, there was recognizable potential for material and technical gain that stood to be made which in turn encouraged the mainstreaming of refugees into national services. The escalating health needs of Syrian refugees brought the country funds that were used to strengthen the national health infrastructure. Politicians, policymakers, and the royal family themselves frequently addressed the international community, making pleas for aid to support strengthening national services including healthcare infrastructure. In a piece written in the Lancet in 2013, the Jordanian Minister of Health pleaded with the “the international community to respond to the many health needs of Syrian refugees, “after which he listed the exact costs needed: “180 million are needed to expand and upgrade ten existing facilities in the northern governorates to cope with the massive demand on health care posed by the Syrian refugees. Additionally, we urgently need a boost to the MOH’s expenditure on health proportional to the numbers of refugees; it should be around $135 million for 2013 and readjusted for 2014 and beyond.” [31] Aid injections quickly followed. In July 2013, an emergency loan in the amount of USD150 million was approved by the World Bank, for the health sector, bread and fuel subsidies, considered some of the most significant services provided to the refugee population [32]. By March 2022, the Bank’s active portfolio in Jordan comprised 14 projects valued at $2.75 billion in loans, concessional financing and grants. In contrast, in 2010, the Bank’s projects were valued at $288.5 million [33].

### Global pressures and international reputation

While the overall national mood to welcome Syrian refugees at the beginning of the crisis was already favorable, Jordan’s desire to earn a reputation as a hospitable country for refugees and to bolster its visibility on the global stage helped sustain commitment for integration. As noted by one policymaker interviewed in the study, Jordan conceded to donor demands and pressures to provide free healthcare to Syrians because it stood to make reputational gains and because doing so would boost its international standing:

“To win political points and a good reputation in the international community, Jordan decided to integrate refugees into the population and treat them like Jordanian citizens. There were so many pressures by the international actors to provide formal healthcare coverage for Syrian refugees.” Policymaker

Increasing donor interest in long-term solutions and global recommendations for mainstreaming refugees residing in urban areas into health systems helped generate momentum for integration [34]. Unfolding on the global agenda was the recognition that forced displacement patterns and trends were changing. Crises were becoming more protracted, forced displacement was becoming increasingly urbanized, and displaced populations from middle-income countries were likely to possess higher baseline incomes, overall better health profiles, and higher levels of non-communicable diseases. Discourse around integration of refugees into local systems was developing. Participants noted that this was key in generating the political will needed to provide refugees with subsidized healthcare through national institutions.

## Policy stream

### Formulation of health sector policies in reaction to the Syrian crisis in Jordan and beyond

As Jordan’s health sector grappled with how to respond to the health needs of the incoming inflow of refugees, other host countries in the region were in a similar situation. UNHCR played a key role in pushing host countries towards mainstreaming refugees into health systems and in mobilizing funding to support these efforts. Illustratively, each of the host countries introduced initial policies that encouraged integration of refugees into the health system. Turkey, the largest host of Syrian refugees, quickly “cleared the way for Syrians to benefit from the hospitals and health care centers free of charge” in 10 cities impacted by the influx [35]. In September 2013, this decree was expanded to enable Syrian refugees access free-of-charge to services across the country and in October 2014, this provisional measure was made permanent. In Egypt, MOH decree 601/ 2012, similarly, granted Syrians access to all MOH health services, while in Lebanon, health services were available in select primary health facilities at subsidized rates [36, 37]. In a context where surrounding countries were crafting policies that were favorable to integration, the Jordanian MOH promulgated a policy in March of 2012 that allowed registered Syrian refugees free access to the MOH’s primary and secondary health services. Unlike neighboring countries which made services available in select facilities and in areas with high concentrations of refugees, the first iteration of the Jordanian policy extended to all public facilities across Jordan. As was the case in other host countries, access was granted primarily through public facilities (and did not include private facilities) and tertiary healthcare presented a challenge given the high costs associated with it. Quickly however, a UNHCR body was established, the Exceptional Care Committee (ECC), to decide on a case-by-case basis whether to approve a request for tertiary care coverage. The ECC based its decision on a set of factors including cost, prognosis, necessity, adequacy of the suggested treatment and financial vulnerability [36].

## Post-2014: regression in policy

While the 2012 policy supported integration of refugees into the national health system, the policy did not survive for too long. In 2014, the MOH repealed the policy and required Syrian refugees to pay the non-insured Jordanian rate when they used all types of health services provided by the MOH. The government justified its decision based on the financial burden that the quickly growing refugee population was placing on the health system. This policy change, however was not the last, and in 2018, the government introduced even harsher changes to the policy, requiring Syrian refugees to pay 80% of the applicable standard pricing for foreigners. According to UNHCR, this entailed out-of-pocket expenses that were 2–5 times higher than for uninsured Jordanians [38].

The gradual curtailment of health services provided to refugees was emblematic of growing popular discontent with the presence of Syrian refugees in the country. Palpable tensions soon ensued between host and refugee communities due to the financial burden posed by the refugee crisis and the tone quickly shifted from reception and “brotherhood” to hostility [26]. In 2013, signaling changes in attitudes towards Syrian refugees, Jordan imposed border closures with Syria, denying entry to Syrian refugees and specifically Palestinian Syrians. Around the same time, the government created the Directorate of Security affairs for the Syrian refugee camps (which was later expanded in 2014 to include all in-camp and out-of-camp refugees and renamed the Directorate of Syrian Refugee Affairs), signaling an increasingly securitized view of Syrian refugees arriving in the country [39]. With mounting pressure on the economy and increasing competition between host and refugee communities over local resources, attitudes towards Syrian refugees quickly worsened and anti-refugee sentiments intensified. In a 2013 poll conducted by the Center for Strategic Studies, a research institute at the University of Jordan, 71% of Jordanian respondents said they opposed allowing more Syrian refugees into the country [40]. In 2016, ahead of a donor conference about the Syria crisis, King Abdullah told the BBC “the psyche of the Jordanian people, I think it’s gotten to a boiling point. Sooner or later, I think, the dam is going to burst.” [41] In another television interview, King Abdullah referred to the burden posed on the health sector: “Our health sector is saturated…..And Jordanians just have had it up to here. I mean we just can’t take it anymore.” [42] Other shifts took place which had direct implications on local integration of Syrian refugees and their access to national health services. Initially, Jordan had a bail out system that allowed refugees to leave camps and reside with relatives who would act as “sponsors” or “guarantors.” Refugees who left refugee camps without the needed approvals could still obtain a Ministry of Interior service card that enabled them to access health services (as well as other public services such as education) [43]. Beginning 2015, the government restricted issuance of service cards to those who illegally left camps, and then shortly after, cancelled the bailout system altogether, preventing refugees from leaving camps legally and locally integrating in host communities.

These policy regressions can be understood against a backdrop of declining political and popular support for the presence of refugees in the country as well as dwindling economic resources to sustain health integration. At the same time, key informants repeatedly cited that despite initial injections of foreign aid into the economy, the international community quickly turned its back on the country, leaving Jordan to shoulder the financial burden of the crisis alone.

Soon after, the international community scaled-up its funding support of the country and was able to get health integration back on the policy agenda. As this was unfolding in the context of Jordan, global efforts to integrate refugees were underway and new financing instruments to support integration were emerging. The World Bank through its Jordan Emergency Health Project approved an exceptional $150 million loan to the country to help “maintain the delivery of primary and secondary health services to poor uninsured Jordanians and Syrian refugees at Ministry of Health facilities” [44]. In mid-2019, an additional $200 million were approved to help support MoH services [45]. In an act of collective action, a multi-donor trust fund was set up to assist the MoH cover costs, with $22.5 million contributed by the United States, Denmark, and Canada [46]. Repeated injections of foreign aid were followed by a decision from the Jordanian government to rollback its 2018 policy allowing Syrian refugees to gain access once again to MoH public hospitals and primary health care centers at the uninsured Jordanian rate. The Government also decided to exempt Syrian refugees once again from paying user fees for maternal and child services provided in its affiliated maternity and childhood centers.

## Discussion

The integration of Syrian refugees into the Jordanian health system quickly moved onto the agenda and a policy allowing refugees to access all public health facilities across the country went into effect at the beginning of the response. What explains the swift policy response and adoption? Our analysis demonstrates that factors at the problem, political and policy stream converged to make this policy solution appealing to policymakers and the public. In the problem stream, infectious disease outbreaks and an increasing burden of non-communicable diseases served as focusing events. Coupled with a large and sudden influx of refugees into urban areas, the reality of the Syrian influx motivated a shift in thinking around how to deliver health services to refugees. In the political stream, popular opinion initially aligned with the government’s strategic interests to leverage the crisis for material and technical support and international visibility. The government’s framing of the Syria crisis and the costs it was incurring as a result galvanized action. Synergies with global-level conversations around health service delivery models motivated by a growing recognition of changing forced displacement patterns and trends helped create a favorable environment for advancing the issue. At the policy level, policy entrepreneurs across several countries impacted by the Syria refugee influx proposed similar solutions and crafted comparable policies that introduced integration, albeit to different degrees.

However, while alignment in all three streams moved integration onto the agenda, changes in the political stream that occurred over time resulted in a series of policy changes. Popular support for reception and integration of Syrian refugees waned over time as tensions developed between host and refugee communities. Foreign funding dwindled and the financial burden shifted onto national budgets. The increasing securitization of Syrian refugees in the country resulted in more stringent encampment policies, which restricted access to health services outside the confines of contained refugee camps. These developments paved the way to the 2014 and 2018 policies which curtailed refugees’ access to the national health system. Efforts from global actors to counteract these policies took the shape of unprecedented injections of foreign aid which significantly expanded the development assistance portfolio in the country. While these injections may have motivated the rollback of the restrictive 2018 policy, it remains to be seen whether the government’s commitment to integration of refugees into national health systems will survive into the future.

Jordan’s experience with integration of Syrian refugees into its health systems illustrates the importance of a conducive political environment in meeting the health needs of refugee populations and underscores the importance of understanding health policies against a backdrop of foreign and security considerations and international diplomacy. It also highlights the interlinkages between refugee integration into health systems and broader integration into social services more generally. Understanding the integration of Syrian refugees into health systems cannot be divorced from understanding the extent of their economic and social integration. Encampment policies, policies that restrict mobility and urban settlement, and policies that prohibit labor participation of refugees run counter to the health integration agenda. This interdependence aligns with much of the theoretical literature around integration which has drawn attention to the muti-dimensional nature of the concept [47–49]. For example, Ager and Strang define integration in terms of four core domains that include not only health but employment, housing, and education [48]. While they unpack the concept of integration in relation to 10 discrete domains, they repeatedly highlight the interdependence of these domains and the policy and practical implications behind their interdependence. Similarly, Kuhlman’s model of integration defines integration in terms of *spatial*, *economic*, *social*, *political*, *legal*, and *psychological integration*. Again, these multiple dimensions are seen as mutually constitutive and reinforcing. Our case study similarly illustrates that integration of refugees into national health systems goes hand in hand with their integration into society at large [49].

As global discourse on integration intensifies, the experience of Jordan with refugee integration raises several important questions. For one, what exactly constitutes integration into health systems? The 2018 policy departed markedly from the original policy that granted Syrian refugees free access to health services, rendering national services financially prohibitive to Syrians. However, the policy still enabled Syrian refugees to receive services through national institutions. As actors advocate for integration, clarity about what constitutes a successful integration policy is key. Is it a policy that grants ‘access’ to services in theory or in practice? Is it a policy that provides refugees health services on par with hosts regardless of the quality and appropriateness of pre-existing health services? How exactly do we define and measure successful integration of refugees into health systems? Moreover, Jordan’s experience with integration brings to the fore questions around financing and the extent to which integration can be a durable solution if clear funding channels are not available. In the absence of clear financial mechanisms established to support integration, government buy-in and a sustainable integration strategy will remain elusive. With universal health care coverage moving high on government agendas, there is a unique opportunity to use UHC as a platform for establishing government commitment to refugee healthcare. To realize this, however, increased financing in health is a critical pre-requisite.

As countries in the region wrestle with how to respond to the protracted Syria crisis which has resulted in several waves of displacement, the case of Jordan offers important insights and lessons learnt. First, it showcases the importance of global commitment to the integration agenda, which facilitated the original adoption of a policy allowing refugees access to health and the reemergence of integration on the government’s agenda after a wave of “backsliding.” At the same time, there is a lot to be learnt from the way the Jordanian government strategically leveraged new and innovating financing mechanisms introduced by the global community and how it successfully galvanized funding in the context of the crisis through regular dialogue with global actors and international diplomacy. Moreover, the case of Jordan underscores the importance of political will and the role political leaders play in addressing tensions that emerge between host and refugee communities, especially in low and middle income settings where resources are scarce and competition over limited resources is high. Finally, the Jordanian experience highlights the dynamism of the policy process and the importance of policy learning.

This case study should be considered in light of some limitations. For one, data collection and analysis focused on policies spanning the years 2012 to 2019. Updates to the policy may have occurred since, though to the author’s knowledge, no changes to the policy have taken place and no developments have occurred that impact the conclusions of this paper. Secondly, because data collection occurred in 2018, there may have been recall bias among our participants. We believe this bias is not substantial given that we triangulated informant accounts with a thorough media and document review that captured past events and situated them in their historical context. Lastly, there are limitations inherent to the Kingdon model. Specifically, the model assumes that policy adoption occurs at one point in time and only after the convergence of three streams that are specified a priori. We mitigated this by using both deductive and inductive codes to capture any departures from the Kingdon’s framing, and were careful to describe the dynamic nature of the policy and the changes that occurred in the aftermath of the original policy.

## Conclusions

Overall, Jordan provides a compelling case study of integration of refugees into national health systems with important learnings for countries wishing to implement the integration agenda. Several enablers, at the problem, policy and political levels, facilitated the quick adoption of the initial policy. Particularly important was the role of the global community and the alignment of the integration agenda with global calls for bridging the humanitarian development nexus. However, Jordan’s experience demonstrates that policy adoption and implementation are dynamic processes that are subject to change based on the wider social and political environment. Clear from this case study is the challenge of maintaining national and public support for the integration agenda in the absence of sustainable funding streams and budgetary commitments to refugee health. Other considerations, including social tensions between refugees and hosts and a securitarian approach to refugee response, undermine the integration agenda. In the case of Jordan, these were counteracted with the humanitarian community stepping up its financial and technical support of the country, illustrating the importance of global action – premised on the idea of responsibility sharing and solidarity – as well as the need for new and innovating funding instruments for refugee health.

### Supplementary Information

** Supplementary Material 1. **

** Supplementary Material 2. **

## Data Availability

Data is available upon reasonable request from the corresponding author.
