# Supplementary material for: The emergence and regression of political priority for refugee integration into the Jordanian health system: an analysis using the Kingdon’s multiple streams model
Source: Confl Health. 2024 Apr 15;18(Suppl 1):30. doi: 10.1186/s13031-024-00588-3 (PMC11017472; doi:10.1186/s13031-024-00588-3)
Supplement: Supplementary file 1 — Supplementary Material 1. [file 13031_2024_588_MOESM1_ESM.docx]

***Supplementary File 1. Electronic Database Search***

- Database: Ovid MEDLINE(R) and Epub Ahead of Print, In-Process & Other Non-Indexed Citations and Daily <1946 to October 10, 2019>​
- Search Strategy: ​
- --------------------------------------------------------------------------------​
- 1     refugee*.ti,ab. (8197) ​
- 2     Refugees/ (9802) ​
- 3     displaced.ti,ab. (29783) ​
- 4     Jordan/ (4031) ​
- 5     Jordan.ti,ab. (4547) ​
- 6     1 or 2 or 3 (46107) ​
- 7     4 or 5 (6700) ​
- 8     6 and 7 (212) ​

***PubMed Search strategy***

"refugees"[MeSH Terms] OR "refugees"[All Fields] OR "refugee"[All Fields]

"Jordan"[MeSH Terms] OR "Jordan"[All Fields]

#1 AND #2

***Scopus Search strategy***

( TITLE-ABS-KEY ( (**Jordan**) )  AND  ( TITLE-ABS-KEY  ( *Syrian***)) AND ( ( TITLE-ABS-KEY ( **refugee** ) )  OR  ( TITLE-ABS-KEY * refugees* ) ) OR ( TITLE-ABS-KEY  (displaced”))
