# Supplementary material for: The emergence and regression of political priority for refugee integration into the Jordanian health system: an analysis using the Kingdon’s multiple streams model
Source: Confl Health. 2024 Apr 15;18(Suppl 1):30. doi: 10.1186/s13031-024-00588-3 (PMC11017472; doi:10.1186/s13031-024-00588-3)
Supplement: Supplementary file 2 — Supplementary Material 2. [file 13031_2024_588_MOESM2_ESM.docx]

**Supplementary file 2**

**Policymaker/stakeholder interview questions (English Version)**

1. Could you briefly describe your main role and responsibilities vis-à-vis refugees in Jordan, given your position as XX (minister/ general director, etc.)?
   1. To what extent do you have direct contact with refugees (e.g., visiting hospitals and/or informal tented settlements)?
2. Given the current health situation in the country, I would like to start with some questions about refugees and COVID-19, before we shift the discussion to the pre-pandemic era.
   1. To what extent has your office organized itself to conduct a COVID refugee response?
   2. What are the key developments in refugee-related policies since the start of the COVID-19 pandemic?
   3. Which organizations would you say are the major players in Covid-19 refugee response?
   4. How do you coordinate with them?
   5. What are the major funding sources (e.g., donors, OOP etc.) for COVID-19 response in refugee communities (for example, for prevention, detection, isolation, and control measures, testing and treatment)?
   6. Are COVID-19 testing and treatment easily accessible for refugees?
   7. Are testing and treatment free to all refugees, covered all or in part through financing agents, or full cost to all?
   8. Does refugees’ access to testing vary regionally?
   9. How do you think the care received by Syrian refugees in Jordan compares to the care local populations receive for COVID-19 (including vaccine deployment)?
   10. Is there anything else you would like to touch upon related to the refugee response during the COVID-19 crisis or the current situation before we move forward to the pre-pandemic era?
3. Let us go back to the pre-COVID period. In your opinion, what were the key health policy developments since the Syrian refugees began arriving here in 2011?
   1. Probe: [questions regarding country-specific policies based on desk research]
   2. What is your opinion on these policy changes?
   3. What do you think prompted these developments?
   4. Who or which organizations do you believe had central roles in these policy changes?
   5. How would you characterize the role of the MoH in these policy shifts?
   6. How do you believe these policy developments affected health services provision?
   7. How do you believe these policy developments have affected front line healthcare providers?
   8. In a few sentences, could you describe, in your opinion, what the most important aspects of [country’s] prior experience with healthcare for refugees are for the current situation?
4. What would you name as the primary sources of financial support for refugee health services in Jordan?
   1. In your view, how effective have these funding systems been?
   2. How has the refugee situation affected government spending on health care?
   3. How do you think donor funding has affected the quality of healthcare facilities can provide:
      1. To refugees?
      2. To locals?
   4. In your view, what are/have been the main challenges in managing healthcare funding? (probe: corruption?)
   5. Did these funding sources change or shift after the COVID-19?
5. In general, how do you think the healthcare received by Syrian refugees in Jordan compares to the healthcare local populations receive?
   1. What changes have you observed in health service delivery for refugees and host populations over time?
   2. What kinds of economic and financial factors do you see as affecting care for each population?
   3. In your professional capacity, what kinds of regional differences, if any, are you aware of when it comes to refugees’ versus local populations’ experience of health care?
   4. What would you say are there local-level conditions or relationships most likely to affect refugees’ healthcare experience?
6. On a scale of zero to ten, with zero being “not integrated at all” and ten being “completely integrated,” to what extent do you think the refugees are integrated into the health system in Jordan?
   1. Could you explain your answer in a few sentences?
   2. What does it mean for refugees to be “integrated” into the Jordanian healthcare system?
7. If you were at an international conference with someone who has the exact same job as you in [Colombia/Bangladesh/other refugee receiving state]. What advice would you give them, given your experience?
8. Is there anything I should have asked you but did not? Would you like to add anything else?

*Thank you very much for your time; we appreciate your participation in this study.*

**Appendix 2:**

**Policymaker/stakeholder interview questions (Arabic Version)**

1-هل بإمكانك أن تصف بإيجاز دورك المهني الرئيسي، وأن تحدد مسؤولياتك المهنية تجاه اللاجئين، نظراً الى منصبك كـ XX (وزير، مدير عام...)؟

ا- الى أي مدى لديك إتصال مباشر مباشر مع اللاجئين؟ (زيارة المستشفيات، المخيمات غير الرسمية...)

2- نظراً إلى الوضع الصحي الراهن في البلاد، لا بد من طرح بعض الأسئلة حول اللاجئين و وباء كوفيد-19 في البداية، قبل العودة الى فترة ما قبل الجائحة.

أ. إلى أي مدى عملتم على تنظيم عملكم للاستجابة لجائحة كوفيد-19 في مجتمعات اللاجئين؟

ب. ما هي أهم التطورات التي طالت السياسات المتعلقة باللاجئين منذ بداية الجائحة ؟

ج. ما هي المنظمات التي برأيكم تلعب دور رئيسي في الاستجابة لجائحة كوفيد-19 في مجتمعات اللاجئين؟

د. كيف يتم التنسيق معهم من قبلكم ؟

ه. ما هي مصادر التمويل الرئيسية (المانحون، النفقات الشخصية...) للاستجابة لجائحة كوفيد-19 في مجتمعات اللاجئين (كالتدابير الوقائية والكشف والعزل والسيطرة والفحص والعلاج)؟

و. هل يمكن للاجئين الوصول بسهولة إلى فحص الكورونا وتلقي العلاج المناسب؟

ز. هل الفحص والعلاج مجانيان لكل اللاجئين، وهل تتم تغطيتهما كليًا أو جزئيًا من قبل الجهات الضامنة؟

ح. هل تتفاوت عملية وصول اللاجئين إلى الفحوصات بين المناطق ؟

ج. كيف تقارنون جودة الرعاية الصحية الخاصة بكوفيد-19 التي يتلقاها اللاجئون السوريون في الاردن، مع الرعاية الصحية عينها، التي يتلقاها المواطنون (بما في ذلك عملية التلقيح)؟

ك. هل هناك أي شيء آخر تودون التطرق إليه فيما يتعلق بالاستجابة إلى حاجات اللاجئين في ظل أزمة كوفيد-19 أو الوضع الحالي قبل التطرق الى فترة ما قبل الجائحة؟

3- بالعودة الى فترة ما قبل الجائحة، ما هي أهم التطورات التي طالت السياسات الصحية منذ بدء وصول اللاجئين السوريين إلى الاردن عام 2011؟

أ. الاستقصاءات والبحوث: [أسئلة تتعلق بالسياسات الوطنية بناءً على بحوث مكتبية]

ب. ما هو رأيكم في هذه التغييرات في السياسات؟

ج. ما هي العوامل برأيكم التي دفعت نحو هذه التغييرات/ التطورات ؟

د. من أو ما هي المنظمات التي تعتقد أن كان لها أدوارًا أساسية في التغييرات /التطورات السياسية هذه؟

ه. كيف تصفون دور وزارة الصحة في هذه التغييرات / التطورات في السياسات الصحية ؟

و. كيف تعتقدون أن هذه التغييرات / التطورات في السياسات أثرت على عملية تقديم الخدمات الصحية؟

ز. كيف تعتقدون أن هذه التغييرات / التطورات في السياسات أثرت على مقدمي الرعاية الصحية في الخطوط الأمامية؟

ح. برأيكم، ما هي أهم جوانب تجربة [البلد] السابقة مع الرعاية الصحية للاجئين مقارنةً بالوضع الحالي؟

4- هل يمكنكم أن تسموا المصادر التمويلية الرئيسية لتأمين الخدمات الصحية للاجئين في الاردن ؟

ا- برأيكم ما مدى فعالية أنظمة التمويل هذه؟

ب- كيف أثر وضع اللاجئين على إنفاق الحكومة على الرعاية الصحية؟

ج- برأيك كيف أثر تمويل المانحين حتى الآن على نوعية خدمات المرافق الرعاية الصحية:

-للاجئين؟

- للسكان المحليين؟

د- من وجهة نظركم، ما هي / كانت التحديات الرئيسية في إدارة تمويل الرعاية الصحية؟ (مثلاً الفساد)؟

ه. هل تغيرت مصادر التمويل هذه بعد جائحة كوفيد-19؟

5- بشكلٍ عام كيف تقارنون بين الرعاية الصحية التي يتلقاها اللاجؤون (السوريون) في الاردن وتلك التي يتلقاها المواطنون؟

ا- ما هي التغييرات التي لاحظتها في مجال تقديم الخدمات الصحية للاجئين و للمواطنون مع مرور الزمن؟

ب- ما هي برأيك العوامل الإقتصادية والمالية المؤثرة في مجال الرعاية الصحية بالنسبة إلى كل فئة سكانية؟

ج- كيف تتفاوت العناية الصحية المقدمة للاجئين وتلك المقدمة للمواطنين حسب المناطق؟

د- هل تعتقد بوجود ظروف على المستوى المحلي يرجح تأثيرها في ما يختبره اللاجئون في مجال الرعاية الصحية؟

6- على مقياس من صفر إلى عشرة، بحيث يكون الصفر "غير مندمجين على الإطلاق" والعشرة "مندمجين تمامًا"، الى أي مدى برأيكم اندمج اللاجئون في النظام الصحي في الاردن؟

ا- هل يمكنكم توضيح اجاباتك ؟

ب- برأيك ماذا تعني عبارة "دمج الاجئين " في نظام الرعاية الصحية الاردني؟

7-إذا كنتم جالسين بجانب نظيركم ، في إحدى المؤتمرات الدولية، في (كولومبيا/ بنغلادش/ أو أي دولة أخرى مضيفة للاجئين) بم تنصحهم نظرا لخبرتك في هذا المجال؟

8-هل من أسئلة أخرى توجب علينا طرحها عليكم ولم نطرحها؟ هل تودون إضافة أي فكرة؟

*شكرا جزيلا على وقتكم، نقدر مشاركتكم في هذه الدراسة.*

**Appendix 3:**

**Health Managers/District level health managers interview questions (English Version)**

1. Can you please briefly describe your job and say how long you have been working here?
   1. How would you describe the work that this facility does?
   2. What populations does this facility serve?
   3. In general, what words would you use to describe your patients?
   4. To what extent do refugees use this facility compared to local populations?
   5. What was your professional experience before coming here?
2. Given the current health situation in the country, I would like to start with some questions about refugees and COVID-19, before we shift the discussion to the pre-pandemic era.
   1. How would you compare the access and utilization of health services for refugees versus hosts during the pandemic?
   2. How is the pandemic affecting routine healthcare?
   3. How do these effects shape your institution’s plans for continuity of routine care during the pandemic for your patients?
   4. How is the financing of the pandemic response affecting the financing of routine care (re-allocation of resources for COVID-19) for refugees and hosts?
   5. What were your strategies for capacity expansion during the pandemic?
      1. Have you considered recruiting healthcare professionals from the refugee population?
      2. If, yes how was your experience so far?
      3. If no, what were your concerns and challenges?
   6. How are care-rationing decisions being made?
      1. In what ways do nationality and displacement status affect care-rationing decisions?
   7. What are the major funding sources for COVID-19 response in refugee communities (for example, for prevention, detection, isolation, and control measures, testing and treatment)?
   8. Are COVID-19 testing and treatment easily accessible for refugees?
   9. Are testing and treatment free to all refugees, covered all or in part through financing agents, or full cost to all?
   10. Is your organization facing challenges specific to refugees?
       1. *If asked, can suggest*: For example, perhaps people do not have mobile phones or internet connectivity for telehealth, or they cannot move freely due to additional measures targeting this group.
   11. Is there anything else you would like to touch on related to the COVID-19 crisis or the current situation before we move forward to the pre-pandemic era?
3. Let us go back to the pre-pandemic period. What was your experience dealing/working with refugees versus local populations?
4. What were the main sources of funding for your facility pre-pandemic?
   1. Are these sources of funding still available?
5. How has the ongoing refugee situation since 2011 influenced the quality of care your facility is able to provide?
   1. To what extent do you see differences in the quality of care you can provide to refugee versus local populations?
   2. In your opinion, what are the main challenges to providing the best possible care to these populations?
   3. Could you describe an example where you were able to overcome a major challenge in this work?
6. *Has the ongoing refugee situation since 2011 affected your facility’s capacity to provide care?*
   1. *How have the resources available to you as a provider changed since the refugee situation began?*
   2. *How has your daily routine shifted since the situation began?*
   3. ***Probes:*** salaries, professional development, workload, relation to medical com (also include in health manager/district manager protocol)
7. In your opinion, what could decision-makers do to better support facilities such as yours?
   1. Probe: decision-makers could be at any level from the facility’s level to the national and international level.
8. On a scale from zero to ten, with zero being “not integrated at all” and ten being “completely integrated,” to what extent do you think the refugees are integrated into the health system in Jordan?
   1. Could you explain your answer in a few sentences?
   2. What does it mean for refugees to be “integrated” into the Jordanian healthcare system?
9. If you were at an international conference with someone who has the exact same job as you in [Colombia/Bangladesh/other refugee receiving state]. What advice would you give them, given your experience?
10. Is there anything that I should have asked you but have not? Would you like to add anything else?

*Thank you very much for your time; we appreciate your participation in this study.*

**Appendix 4:**

**Health Managers/District level health managers interview questions (Arabic Version)**

1- هل يمكنكم وصف وظيفتكم بإيجاز وذكر مدة استلامكم هذه الوظيفة

أ. كيف تصف العمل الذي تقوم به هذه المنشأة؟

ب. ما هي الفئات التي تخدمها هذه المؤسسة؟

ج. بشكل عام، ما الكلمات التي قد تستخدموها لوصف مرضاكم؟

د. إلى أي مدى يستخدم اللاجئون هذا المرفق مقارنة بالمواطنين؟

ه. ما هي خبرتك المهنية السابقة؟

2- نظراً إلى الوضع الصحي الراهن في البلاد، نود أن نبدأ ببعض الأسئلة حول اللاجئين وجائحة كوفيد-19، قبل أن نعود الى فترة ما قبل الجائحة.

أ. كيف تقارن الوصول إلى الخدمات الصحية واستخدامها من قبل الاجئين مقابل الخدمات الصحية المقدمة للمواطنين أثناء الجائحة؟

ب. كيف يؤثر الوباء على الرعاية الصحية الروتينية ؟

ج. كيف يؤثر الوباء على خطط مؤسستكم لاستمرارية الرعاية الروتينية لمرضاكم أثناء الجائحة؟

د. كيف يؤثر التمويل للاستجابة للوباء على تمويل الرعاية الروتينية (إعادة تخصيص الموارد لكوفيد-19) للاجئين والمواطنين؟

ه. ما هي استراتيجياتكم لتوسيع قدرات مؤسستكم أثناء الوباء؟

* هل فكرتم في توظيف متخصصين في الرعاية الصحية من بين اللاجئين؟

إذا ، نعم كيف كانت تجربتكم حتى الآن؟

إذا كانت الإجابة "لا" ، فما هي المخاوف والتحديات؟

و. كيف يتم اتخاذ قرارات ترشيد خدمات الرعاية الصحية ؟

* كيف تؤثر الجنسيات الوطنية والنزوح على قرارات ترشيد خدمات الرعاية الصحية؟

ز. ما هي مصادر التمويل الرئيسية للاستجابة لوباء كوفيد-19 بين اللاجئين (مثلاً، للوقاية والكشف والعزل والاحتواء والفحص والعلاج)؟

ح. هل يمكن للاجئين الوصول بسهولة إلى فحوصات وعلاج كوفيد-19؟

* هل الفحص والعلاج مجانيان لجميع اللاجئين، و هل يتم تغطيتهما كليًا أو جزئيًا من قبل الجهات الضامنة؟

ي. هل تواجه مؤسستكم تحديات خاصة باللاجئين؟

* **إذا طُلب منك ذلك، يمكن أن يقترح:** مثلاً، ربما لا يمتلك بعض الأشخاص هواتف محمولة أو اتصالاً بالإنترنت للخدمات الصحية عن بُعد، أو لا يمكنهم التنقل بحرية بسبب التدابير الإضافية التي تستهدف هذه الفئات.

ك. هل هناك أي موضوع آخر تود التطرق إليه فيما يتعلق بأزمة كوفيد-19 أو الوضع الراهن، قبل التطرق الى فترة ما قبل الجائحة؟

3- بالعودة الى فترة ما قبل الجائحة، ما هي تجربتكم في التعامل / العمل مع اللاجئين مقارنةً بالمواطنين؟

4- ما هي المصادر التمويلية الرئيسية لمنشأتكم خلال فترة ما قبل الوباء؟

أ. هل مصادر التمويل هذه لا تزال متاحة؟

ب- ما هي برأيك التحديات الأساسية في وجه تأمين أفضل خدمات الرعاية الممكنة لهذه الشرائح السكانية؟

ث- هل يمكنك أن تعطينا مثلا تشرح من خلاله كيف تمكنت من تخطي أحد التحديات الكبرى في مجال عملك هذا؟

5- كيف أثر وضع اللاجئين المستمر منذ عام 2011 على جودة الرعاية الصحية التي يمكن لمرفقكم الصحي تقديمها؟

أ. إلى أي مدى ترون الاختلافات في جودة الرعاية الصحية التي يمكنكم تقديمها للاجئين مقارنةً بالمواطنين؟

ب. برأيكم، ما هي التحديات الرئيسية لتقديم أفضل رعاية ممكنة لهؤلاء للاجئين والمواطنين ؟

ج. هل يمكنكم وصف مثال تمكنتم من خلاله على التغلب على تحد كبير في عملكم؟

6- ماذا بوسع صناع القرار القيام به لدعم مرافق مماثلة لمرفقكم بشكل أفضل؟ هل أثر وضع اللاجئين المستمر منذ عام 2011 على قدرة مؤسستكم على تقديم الرعاية؟

أ. كيف تغيرت الموارد المتاحة لك كمقدم للرعاية الصحية منذ بداية اللجوء؟

ب. كيف تغير روتينكم اليومي منذ أن بدأت موجة اللجوء؟

ج. **استقصاء:** الرواتب، التطوير المهني، والأعباء العملية والعلاقة البروتوكولية بين المدير الصحي والمسؤولين عن المنطقة.

7- برأيكم، ما الذي يمكن أن يقوم به صانعو القرار لتعزيز دعم المرافق المماثلة لمرفقكم؟

أ. *الاستقصاء*: يمكن أن يكون صناع القرار، على مستوى المنشأة إلى المستوى الوطني والدولي.

8- على مقياس من صفر إلى عشرة، مع كون الصفر "غير مندمجين على الإطلاق" والعشرة "مندمجين تمامًا" ، إلى أي مدى تعتقد أن اللاجئين قد اندمجوا في النظام الصحي في الاردن؟

أ. هل يمكن أن تشرح إجابتكم ؟

ب. ماذا يعني "اندماج" اللاجئين في نظام الرعاية الصحية الاردني؟

9- إذا كنتم في مؤتمر دولي مع نظيركم [كولومبيا / بنغلاديش / دولة أخرى لاستقبال اللاجئين]. ما هي النصيحة التي تقدموها لهم، بحسب خبرتكم؟

10- هل من أسئلة أخرى توجب علي طرحها عليكم ولم أطرحها ؟ هل تودون إضافة أي فكرة؟

*شكرا جزيلا على وقتكم، نقدر مشاركتكم في هذه الدراسة.*

**Appendix 5:**

**Health workers interview questions (English Version)**

1. Can you please briefly describe your job and say how long you have been working here?
   1. How would you describe the work that this facility does?
   2. Which populations do you serve? In general, who are your patients?
   3. To what extent do you have direct contact with refugees?
   4. *For refugee health providers*: What was your professional experience before coming here?
   5. *For refugee health providers*: What are some of the similarities and differences of practicing in your home country versus here?
   6. *For refugee health providers*: *Can you name one or two specific events or share with me one or two stories that you feel represent your experience as a healthcare provider here in Jordan?*
   7. *For host country health providers:* Have you experienced displacement in your own life? If yes, *what was your experience of healthcare during your displacement?*
2. Given the current health situation in the country, I would like to start with some questions about refugees and COVID-19, before we shift the discussion to the pre-pandemic era.
   1. How would you compare the access and utilization of health services for refugees versus hosts during the pandemic?
   2. How are care-rationing choices being made?
      1. In what ways do nationality and displacement status affect care-rationing decisions?
   3. How has the pandemic affected routine healthcare?
      1. How does this affect your institution’s plan for continuity of routine care during the pandemic for your patients?
   4. Are your refugee patients facing an specific challenges?
      1. *If asked, can suggest:* For example, perhaps people do not have mobile phones or internet connectivity for telehealth, or they cannot move freely due to additional measures targeting this group.
   5. Are COVID-19 testing and treatment easily accessible for refugees?
   6. Are testing and treatment free to all refugees, covered all or in part through insurance if people have it, or full cost to all?
   7. Is there anything else you would like to touch on related to the COVID-19 crisis or the current situation before we move forward to the pre-pandemic era?
3. Let us go back to the pre-pandemic period. What was your experience treating refugees versus local populations?
4. How has the ongoing refugee situation since 2011 influenced the quality of care you are able to provide?
   1. To what extent do you see differences in the quality of care you can provide to refugee versus local populations?
   2. In your opinion, what are the main challenges to providing the best possible care to these populations?
   3. Could you describe an example where you were able to overcome a major challenge in this work?
5. Has the refugee situation since 2011 affected your facility’s capacity to provide care?
   1. How have the resources available to you as a provider changed since the refugee situation began?
   2. How has your daily routine shifted since the situation began?
   3. ***Probes:*** salaries, professional development, workload, relation to medical com (also include in health manager/district manager protocol)
   4. *In a few words, can you describe how the professional situation of people in your position has changed since the refugee situation began? (Probes: new services, new resources, training opportunities)*
6. In your opinion, what could decision-makers do to better support front-line healthcare providers?
   1. Probe: decision-makers could be at any level from the facility’s level to the national and international level.
7. Is there anything that I should have asked you but have not? Would you like to add anything else?

*Thank you very much for your time; we appreciate your participation in this study.*

**Appendix 6:**

**Health workers interview questions (Arabic version)**

1- هل يمكنكم وصف مهماتكم الوظيفية بإيجاز وذكر مدة استلامكم هذه الوظيفة ؟

أ. كيف تصف العمل الذي تقومون به في هذه المنشأة؟

ب. ما هي الفئات السكانية التي تخدموها؟ بشكل عام، من هم مرضاكم؟

ج. إلى أي مدى تتصلون بشكل مباشر مع اللاجئين؟

د. لمقدمي خدمات الصحة للاجئين: ما هي خبرتكم المهنية السابقة؟

ه. بالنسبة لمقدمي خدمات الرعاية الصحية للاجئين: ما هي بعض أوجه التشابه والاختلاف بين الممارسة في بلدك مقابل الممارسات هنا؟

و. لمقدمي خدمات الرعاية الصحية للاجئين: هل يمكنكم تسمية حدث أو حدثين محددين أو مشاركة قصة أو قصتين تشعر أنهما تمثلان تجربتكم كمقدم رعاية صحية هنا في الاردن؟

ز. لمقدمي الخدمات الصحية في البلد المضيف: هل اختبرتم النزوح في حياتكم؟ إذا كانت الإجابة بنعم، فما هي تجربتكم في الرعاية الصحية أثناء النزوح؟

2- بالنظر إلى الوضع الصحي الراهن في البلاد، نود أن نطرح بعض الأسئلة حول اللاجئين و كوفيد-19، قبل أن نعود إلى فترة ما قبل الجائحة.

أ. كيف تقارنون الوصول إلى الخدمات الصحية للاجئين مقابل المواطنين أثناء الجائحة؟

ب. كيف يتم اتخاذ خيارات ترشيد خدمات الرعاية الصحية؟

* ما هي الطرق التي تؤثر بها الجنسيات الوطنية والنزوح على قرارات ترشيد خدمات الرعاية الصحية؟

ج. كيف أثر الوباء على الرعاية الصحية الروتينية؟

* كيف يؤثر ذلك على خطة مؤسستكم لاستمرار الرعاية الروتينية لمرضاكم أثناء الجائحة؟

د. هل يواجه مرضاكم اللاجئون تحديات معينة؟

* *إذا طُلب منكم ذلك، يمكن الاقتراح*: مثلاً، ربما لا يمتلك الأشخاص هواتف محمولة أو اتصالاً بالإنترنت للحصول على خدمات الصحية عن بُعد، أو لا يمكنهم التنقل بحرية بسبب التدابير الإضافية التي تستهدف اللاجئين.

ه. هل يمكن للاجئين الوصول بسهولة إلى فحص وعلاج كوفيد-19؟

و. هل الفحص والعلاج مجانيان لجميع اللاجئين، ويتم تغطيتهما كليًا أو جزئيًا من من قبل الجهات الضامنة؟

ز. هل هناك أي شيء آخر تودون التطرق إليه فيما يتعلق بأزمة كوفيد-19 أو الوضع الراهن قبل العودة الى فترة ما قبل الجائحة؟

3- بالعودة الى فترة ما قبل الجائحة، كيف أثرت الحالة الراهنة في نوعية الرعاية التي تستطيع تقديمها؟

أ- إلى أي درجة ترى أوجه اختلاف في جودة الرعاية التي تستطيع تقديمها للاجئين مقابل تلك المقدمة إلى السكان المحليين؟

ب- ما هي برأيك التحديات الرئيسية لتقديم أفضل أشكال الرعاية الممكنة لهذه الفئات السكانية؟

ج- هل يمكنك أن تعطينا مثلا تصف من خلاله كيف تمكنت من تخطي أحد التحديات الكبرى في مجال عملك هذا؟

4- كيف أثر وضع اللاجئين المستمر منذ عام 2011 على جودة الرعاية التي يمكنكم تقديمها؟

أ. إلى أي مدى ترون الاختلافات في جودة الرعاية التي يمكنك تقديمها للاجئين مقابل الرعاية عينها المقدمة للمواطنين؟

ب. برأيكم، ما هي التحديات الرئيسية لتقديم أفضل رعاية ممكنة للاجئين؟

ج. هل يمكنكم وصف مثال تمكنتم فيه من التغلب على تحد كبير في عملكم؟

5- هل أثر وضع اللاجئين منذ عام 2011 على قدرة منشأتكم على تقديم الرعاية؟

أ. كيف تغيرت الموارد المتاحة لكم كمقدم رعاية صحية منذ بدء موجة اللجوء؟

ب. كيف تغير روتينكم اليومي منذ أن بدأت موجة اللجوء؟

ج. **تحقيقات:** الرواتب، والتطوير المهني، والأعباء الوظيفية، والعلاقة بالمدير الصحي / والمسؤول عن المنطقة)

د. باختصار، هل يمكنكم أن تصفوا كيف تغير الوضع المهني للأشخاص الذين شغلوا منصبكم منذ بدء موجة اللجوء؟ (تحقيقات: خدمات جديدة، موارد جديدة، فرص تدريب)

6- برأيكم، ما الذي يمكن أن يقوم به صناع القرار لدعم مقدمي الرعاية الصحية في الخطوط الأمامية بشكل أفضل؟

أ. التحقيق: يمكن أن يكون صناع القرار على أي مستوى من مستوى المنشأة إلى المستوى الوطني والدولي

7- هل من أسئلة أخرى كان علينا طرحها عليكم ولم نطرحها ؟ هل تودون إضافة أي شيء؟

*شكرا جزيلا لوقتكم، نقدر مشاركتكم في هذه الدراسة.*

**Appendix 7:**

**Patient focus group discussion guide (English Version)**

*Start with introduction of enumerator, introduction of each participant, consent procedures, etc.*

1. To start, could each of you share three words that reflect your experience of healthcare services here before the pandemic started? [*Enumerator writes every word on board*]
2. Could you now please share another three words that reflect your experience of healthcare services specifically since the COVID-19 pandemic started? [*Enumerator writes every word on board*]
3. Would someone like to pick one of these words and to briefly share how it represents one of their experiences with healthcare services? [Enumerator circles word]
4. Have other people here had similar experiences?
5. Does anyone have an experience that contrasts these experiences?
6. [prompt to move on after a few people speak]
7. Would someone like to pick a second word and to share an experience related to that word? [*Enumerator circles word*]
8. Have other people here had similar experiences?
9. Does anyone have an experience that is different from these experiences?

[*prompt to move on after a few people speak*]

[*if overwhelmingly negative words chosen, can constructively suggest a third more subjectively “positive” word that was offered to repeat process a third time*]

1. Now, I’d like you to think about an average visit to a healthcare provider. Can you please share 2 similarities and 2 differences between:

*For refugee*: A visit to a healthcare provider before you came to Jordan and a visit to a healthcare provider here in Jordan today?

*For host community*: A visit to a health provider before the Syrian refugee situation began in 2011 and today.

- 1. Could you describe any broad changes to health services that you have noticed over time [Probe on similarities and differences: Access issues, quality issues, financial, waiting times, availability of medications, availability of specialists, different localities/facilities/cross-regional]
  2. Do you feel that anything has changed specifically because of the pandemic?
  3. How do you feel this has affected the quality of care that you receive?
  4. How do you feel this has affected your overall access to healthcare?

1. I’m going to give you four potential scenarios. I would like you to share with us what you would tell the woman to think about as she decides how to seek care and where to go.
   1. In the first scenario, a woman has been hit by a car and has a large cut on her face. The bleeding is very bad and will not stop. What would you advise her to do?
   2. In this second scenario, a woman is always very tired, has frequent headaches, and generally feels down and depressed. What would you advise her to do?
   3. In this third scenario, a woman has an infant who has had a 40 degrees fever for three days. What would you advise her to do?
   4. In the fourth scenario, a woman is feeling very sick, has high fever and shortness of breath, and recently lost her ability to smell and taste. What would you advise her to do?
2. What do you think matters most in getting quality healthcare in Jordan?
3. Based on the discussion we’ve just had, and your broader experience, what one piece of advice you would give to decision-makers and health care providers?
4. What changes would you like to see to healthcare service provision in Jordan?

*Does anyone have a brief final thought to add before we finish? Thank you so much for your time.*

**Appendix 8:**

**Patient focus group discussion guide (Arabic Version)**

تستهل المقابلة بتقديم القائم بالتعداد والمشاركين فرداً فرداً وبإجراءت الموافقة إلخ

1. في البداية هل يمكن لكل منكم طرح ثلاث كلمات تعكس تجربته في ما يتعلق بخدمات الرعاية الصحية هنا قبل بدء الجائحة ؟

)*تكتب كل كلمة على اللوح* (

أ- هل يرغب أحدكم في إختيار إحدى هذه الكلمات لتمثل بإيجاز تجربة مر بها في مجال خدمات الرعاية الصحية ؟(يضع ألقائم بالتعداد دائرة حول هذه الكلمة )

ب -هل يوجد أشخاص اخرون هنا سبق أن مروا بتجارب مماثلة ؟

ج- هل لدى البعض منكم تجريا مختلفة؟

د - (بعد تكلم بعضهم عن تجاربه بدر بالإنتقال إلى السؤال التالي )

ه-هل يرغب أحدكم في إختيار كلمة ثانية تمثل تجربة مر بها بهدف تبادل التجارب ؟(يضع ألقائم بالتعداد دائرة حول الكلمة)

و- هل لدى بعد منكم تجارب مماثلة؟

ز - هل مرة أحداً منكم بتجربة مختلفة؟

)بادر بالإنتقال إلى الخطوة التالية(

د- في حال كانت الكلمات المختارة سلبية في معظمها، يمكن إقتراح كلمة ثالثة ،بطريقة بناءة ، تنطوي على معنى "إيجابي" ، وإتباع الطريقة ذاتها مرة ثالثة

2- هل يمكنكم الآن مشاركة ثلاث كلمات أخرى تعكس تجربتكم في خدمات الرعاية الصحية منذ أن بدأ جائحة كوفيد-19؟ *تكتب كل كلمة على اللوح*

3) هل يرغب أحدكم في اختيار إحدى هذه الكلمات ومشاركة باختصار كيف تمثل إحدى تجاربهم في تقديم خدمات الرعاية الصحية؟

أ. هل كان لأشخاص آخرين هنا تجارب مماثلة؟

ب. هل لأي شخص خبرة تتناقض مع هذه التجارب؟

ج. [موجه للمضي قدمًا بعد أن يتحدث عدد قليل من الأشخاص]

د. هل يرغب شخص آخر في اختيار كلمة ثانية ومشاركة تجربة تتعلق بهذه الكلمة؟

ه. هل كان لدى أشخاص آخرين هنا تجارب مماثلة؟

و. هل لدى أي شخص تجربة مختلفة عن هذه التجارب؟

[موجه للمضي قدمًا بعد أن يتحدث عدد قليل من الأشخاص]

إذا تم اختيار الكلمات السلبية بأغلبية ساحقة، فيمكن أن تقترح بشكل بناء كلمة ثالثة "إيجابية" أكثر ذاتية والتي تم عرضها لتكرار العملية مرة ثالثة]

4) الآن، نود أن تفكروا في زيارة عادية لمقدم الرعاية الصحية. هل يمكنكم مشاركة اثنين من أوجه تشابه و اثنين من الاختلافات بين:

للاجئين: زيارة لمقدم رعاية صحية قبل مجيئكم إلى الاردن وزيارة أحد مقدمي الرعاية الصحية هنا في الاردن اليوم؟

للمجتمع المضيف: زيارة أحد مقدمي الخدمات الصحية قبل بدء موجة اللجوء عام 2011 واليوم.

أ) هل يمكنك وصف أي تغييرات جذرية في الخدمات الصحية التي لاحظتها عبر مرور الوقت [دقق في أوجه التشابه والاختلاف: مشكلات الوصول الى الخدمات، ومشكلات جودة الخدمات، وأوقات النتظار، وتغييرات على الصعيد المالي، وتوافر الأدوية، وتوافر المتخصصين، والمواقع / المرافق المختلفة / عبر المناطق]

ب) هل تشعر أن أي شيء قد تغير على وجه التحديد بسبب الوباء؟

ج) كيف تشعر أن هذا الأمر أثر على جودة الرعاية التي تتلقاها؟

د) كيف تشعر أن هذا الأمر أثر على وصولك بشكل عام إلى الرعاية الصحية؟

5- سنطرح أمامكم أربعة سيناريوهات محتملة. نود أن تشاركنا ما ستقوله للمرأة للتفكير فيه وهي تقرر كيفية طلب الرعاية وإلى أين تذهب.

أ) في السيناريو الأول، صدمت امرأة من قبل سيارة وسبب الحادث جرح كبير في وجهها. النزيف سيء جدا لا يتوقف. ماذا تنصحها أن تفعل؟

ب) في السيناريو الثاني، تعاني المرأة من تعب دائم، وتعاني من صداع متكرر، وتشعر عمومًا بالإحباط والاكتئاب. ماذا تنصحها أن تفعل؟

ج) في السيناريو الثالث، يعاني رضيع امرأة من حمى 40 درجة لمدة ثلاثة أيام. ماذا تنصحها أن تفعل؟

د) في السيناريو الرابع، تكون المرأة مريضة للغاية وتعاني من ارتفاع في درجة الحرارة وضيق في التنفس وفقدت مؤخرًا قدرتها على الشم والتذوق. ماذا تنصحها أن تفعل؟

6- ما هو برأيكم أكثر الأمور أهمية في الحصول على رعاية صحية جيدة في الاردن؟

7- بناءً على المناقشة التي أجريناها للتو، وخبرتكم الأوسع، ما النصيحة التي ستقدموها لصانعي القرار ومقدمي الرعاية الصحية؟

8- ما هي التغييرات التي تود أن تراها في عملية تقديم خدمات الرعاية الصحية في الاردن؟

هل من فكرة أخيرة يود أحدكم اضافتها إلى ما سبق قبل الختام ؟ شكراً *جزيلاً على وقتكم.*
